# Novel Combined Immune Deficiency and Radiation Sensitivity Blended Phenotype in an Adult with Biallelic Variations in *ZAP70* and *RNF168*

**DOI:** 10.3389/fimmu.2017.00576

**Published:** 2017-05-26

**Authors:** Ivan K. Chinn, Robert P. Sanders, Asbjørg Stray-Pedersen, Zeynep H. Coban-Akdemir, Vy Hong-Diep Kim, Harjit Dadi, Chaim M. Roifman, Troy Quigg, James R. Lupski, Jordan S. Orange, I. Celine Hanson

**Affiliations:** ^1^Department of Pediatrics, Baylor College of Medicine, Houston, TX, USA; ^2^Section of Immunology, Allergy, and Rheumatology, Texas Children’s Hospital, Houston, TX, USA; ^3^Center for Human Immunobiology, Texas Children’s Hospital, Houston, TX, USA; ^4^Texas Transplant Institute, Methodist Hospital, San Antonio, TX, USA; ^5^Norwegian National Unit for Newborn Screening, Department of Pediatric and Adolescent Medicine, Oslo University Hospital, Oslo, Norway; ^6^Institute of Clinical Medicine, University of Oslo, Oslo, Norway; ^7^Baylor-Hopkins Center for Mendelian Genomics, Baylor College of Medicine, Houston, TX, USA; ^8^Department of Molecular and Human Genetics, Baylor College of Medicine, Houston, TX, USA; ^9^Division of Immunology and Allergy, Department of Pediatrics, The Hospital for Sick Children, University of Toronto, Toronto, ON, Canada; ^10^Canadian Centre for Primary Immunodeficiency, The Jeffrey Model Research Laboratory for the Diagnosis of Primary Immunodeficiency, The Hospital for Sick Children, University of Toronto, Toronto, ON, Canada; ^11^Human Genome Sequencing Center, Baylor College of Medicine, Houston, TX, USA

**Keywords:** primary immunodeficiency, *ZAP70*, *RNF168*, radiosensitivity, immunodeficiency, dysmorphic features, and learning difficulties syndrome, whole-exome sequencing, blended phenotype

## Abstract

With the advent of high-throughput genomic sequencing techniques, novel genetic etiologies are being uncovered for previously unexplained Mendelian phenotypes, and the underlying genetic architecture of disease is being unraveled. Although most of these “mendelizing” disease traits represent phenotypes caused by single-gene defects, a percentage of patients have blended phenotypes caused by pathogenic variants in multiple genes. We describe an adult patient with susceptibility to bacterial, herpesviral, and fungal infections. Immunologic defects included CD8^+^ T cell lymphopenia, decreased T cell proliferative responses to mitogens, hypogammaglobulinemia, and radiation sensitivity. Whole-exome sequencing revealed compound heterozygous variants in *ZAP70*. Biallelic mutations in *ZAP70* are known to produce a spectrum of immune deficiency that includes the T cell abnormalities observed in this patient. Analyses for variants in genes associated with radiation sensitivity identified the presence of a homozygous *RNF168* variant of unknown significance. *RNF168* deficiency causes radiosensitivity, immunodeficiency, dysmorphic features, and learning difficulties syndrome and may account for the radiation sensitivity. Thus, the patient was found to have a novel blended phenotype associated with multilocus genomic variation: i.e., separate and distinct genetic defects. These findings further illustrate the clinical utility of applying genomic testing in patients with primary immunodeficiency diseases.

## Introduction

### Clinical Presentation

This individual has been reported within a larger cohort study (subject 27.1), and we now describe the detailed clinical and genomic information concerning the case (1). The patient is a 30-year-old female of Mexican-American descent who presented with a clinical history of susceptibility to infections that suggested the presence of an underlying primary immunodeficiency disease (PIDD). She developed recurrent otitis media and upper respiratory tract infections starting at 3 months of age, and from 3 years of age she had difficulty with severe, recurrent warts. Repeated episodes of pneumonia began at the age of 7 years, resulting in chronic lung disease and bronchiectasis. At the age of 10 years, she developed chickenpox, which was noted to be more severe than in her sisters, who had concurrent symptoms. At the age of 11 years, she was diagnosed with cryptococcal meningitis. She was treated with fluconazole for several weeks as an inpatient and 4 months as an outpatient. Upon discontinuation of treatment, severe, recurrent episodes of meningitis rapidly ensued, resulting in hydrocephalus and ventriculo-peritoneal (VP) shunt placement. This complication was associated with damage of her vision, rendering her legally blind. The patient was then placed on daily fluconazole and intravenous immunoglobulin (IVIG) prophylaxes and subsequently demonstrated persistent serum cryptococcal antigen positivity but no clinical disease or further life-threatening illnesses. In adult life, she developed recurrent human papilloma virus (HPV)-associated oral and cutaneous lesions. She also developed low-grade squamous intraepithelial lesions of the cervix that were attributed to HPV infection. The cervical dysplasia required localized surgical resection. Due to poor response of the other HPV-associated lesions to topical antiviral therapy, interferon-gamma (IFN-γ) treatment was initiated, producing resolution of the oral disease. The cutaneous problems persisted, however. Many of these lesions transformed to squamous cell carcinomas, causing disfigurement due to numerous resections and destructive topical treatments. Treatment for the progressive skin disease included an option for combined surgery and radiotherapy, which was deferred due to the absence of a specific immunologic diagnosis.

### Laboratory Test Results

At the time of the cryptococcal meningitis diagnosis, the patient was evaluated by an immunologist outside of our institutions and diagnosed with combined immunodeficiency characterized by low serum IgG levels (Table 1) and T cell deficiency. DNA PCR testing excluded HIV infection. Although she was subsequently placed and maintained on IVIG replacement, no further immunologic testing was performed until she was transferred to our care at 21 years of age. At that time, she began to receive regular immunologic evaluations, and the T cell defects were observed to have persisted (Table 1). Immunophenotyping demonstrated variable T cell lymphopenia, predominantly involving the CD8^+^ subset (2). T cell proliferative responses were measured using isolated peripheral blood mononuclear cells cultured in microwell plates loaded with 10-fold dilutions of mitogens or specific antigens. The responses were enumerated in terms of counts per minute (cpm) of tritiated thymidine incorporation. These tests showed decreased T cell activity toward mitogens, particularly at lower concentrations. The responses to antigens were very low, as well, but could not be interpreted due to lack of recent immunization (on IgG supplementation) or exposure (to *Candida*). Evaluation of natural killer (NK) cell function showed abnormal CD107a mobilization, suggesting poor NK cell degranulation (Table 1).

**Table 1:** **Immunologic studies in a patient with *ZAP70* and *RNF168* variants**.

	Normal reference					
Age (years)		11	21	27	28	29
**Immunoglobulins**						
IgG (mg/dL)	641–1,353	<400	1,020^a^	1,320^a^	1,534^a^	1,375^a^
IgA (mg/dL)	66–295		111	102	ND	ND
IgM (mg/dL)	40–180		124	94	ND	ND
IgE (IU/mL)	0–100		ND	<1	ND	<4
**Lymphocyte subsets**						
Lymphocytes (cells/mm^3^)			952	1,058	609	1,023
Total CD3^+^ (cells/mm^3^)	798–2,594		750	1,342	524	952
CD3^+^CD4^+^ (cells/mm^3^)	579–1,841		604	1,049	443	508
CD3^+^CD8^+^ (cells/mm^3^)	184–855		84	205	55	133
CD4^+^:CD8^+^ T cell ratio	1.13–3.5		7.2	5.1	8.1	3.8
CD16^+^/CD56^+^ (cells/mm^3^)	89–472		119	128	12	152
CD19^+^ (cells/mm^3^)	63–461		134	126	49	95
**Proliferation testing (cpm)**						
PHA (10 µg/mL)	163,507–415,087		47,124	62,544	82,419	ND
PHA (1.0 µg/mL)	35,494–225,107		338	4,405	ND	ND
ConA (50 µg/mL)	80,718–286,866		14,454	83,446	ND	ND
ConA (5.0 µg/mL)	28,998–108,585		101	29,298	ND	ND
PWM (100 ng/mL)	37,006–157,955		15,176	39,050	ND	ND
PWM (10 ng/mL)	24,369–94,311		1,271	11,994	ND	ND
*Candida* antigen	≥2,000		2	209	71	ND
*Diphtheria* antigen	≥2,000		ND	48	ND	ND
Tetanus antigen	≥2,000		8	0	ND	ND

**Advanced testing**	**Normal reference**	**Patient result**				

CD107a mobilization assay						
Age (years)		26				
CD107a% expression	11–35%	5%				
CD107a median fluorescence intensity	207–678	121				
**Colony survival assay**						
Age (years)		28				
Patient survival fraction	37–63%	8%				
Radiosensitive range	7–21%					

*^a^On IgG supplementation*.

*ConA, concanavalin A; ND, not tested; PHA, phytohemagglutinin; PWM, pokeweed mitogen*.

At 28 years of age (Table 1), as part of the ongoing immunologic evaluation, the patient was tested for a DNA repair disorder in a College of American Pathologists accredited and Clinical Laboratory Improvement Amendments certified laboratory (Diagnostic Molecular Pathology and DNA Repair Clinical Testing Laboratory, University of California at Los Angeles) (3, 4). Briefly, peripheral blood lymphocytes were immortalized *in vitro* with Epstein-Barr virus. Once a persistent lymphoblastoid cell culture (LCL) was established, various concentrations of cells were plated in 96-well plates. Some of these plates were irradiated with 1 Gy, while others were kept as controls. After 2 weeks of culture, the number of surviving colonies in each plate was enumerated to determine a survival fraction. Positive and negative controls were included with each testing batch. The test revealed the presence of significant radiation sensitivity (8% survival fraction for patient LCLs), a percentage typically seen in patients with ataxia-telangiectasia.

Several diagnostic possibilities were considered at this point. The CD8^+^ T cell lymphopenia and decreased T cell proliferative responses to mitogens suggested the presence of zeta chain-associated protein of 70 kiloDaltons (ZAP70) deficiency, although the presence of hypomorphic mutations in severe combined immunodeficiency disease (SCID)-causing genes or a novel genetic defect could not be immediately excluded as possible explanations (5–8). Although ZAP70 deficiency could account for the T cell defects present in the patient, it is not known to cause radiation sensitivity, and the protein is not associated with any DNA repair pathways. On the other hand, mutations in several SCID-causing genes (i.e., *DCLRE1C, LIG4, PRKDC*, and *NHEJ1*) are known to cause radiosensitivity and could produce a unifying diagnosis. Meanwhile, other genes associated with radiosensitivity and immune deficiency merited consideration and included *ATM, NBN, MRE11A, RAD50, APTX, BLM, DKC1*, and *RNF168* (9). In fact, several of these genetic defects have been categorized together to form the clinical entity known as XCIND syndrome, which is characterized by *X*-ray hypersensitivity, *c*ancer susceptibility, *i*mmunodeficiency, *n*eurological abnormality, and *d*ouble-strand DNA breakage (10–12). Upon further testing, however, the patient was determined to have normal protein levels of ataxia-telangiectasia mutated (ATM); nibrin; meiotic recombination 11, *S. cerevisiae* homolog of, A (MRE11); RAD50; DNA ligase 4; and aprataxin by western blotting. Normal enzymatic kinase activity of the ATM protein was also confirmed.

Thus, written informed consent was obtained from the patient and her family members to participate in a Baylor College of Medicine Institutional Review Board approved protocol for whole-exome sequencing (WES) as part of the Baylor-Hopkins Center for Mendelian Genomics project at Baylor College of Medicine (Houston, TX, USA). The patient and family members also provided written informed consent to have their clinical and genetic information published in medical or scientific journals. All procedures performed in studies involving human participants were in accordance with the ethical standards of the institutional and/or national research committee and with the 1964 Helsinki declaration and its later amendments or comparable ethical standards. WES was performed by the Baylor College of Medicine Human Genome Sequencing Center (BCM-HGSC) using genomic DNA extracted from whole blood. Sequencing was performed with greater than 90% coverage at a read-depth of 20× or greater. Stratified disease-associated variants were confirmed by Sanger sequencing. Methodology, CORE design, and variant selection have been previously described (1, 13).

**Figure 1:**
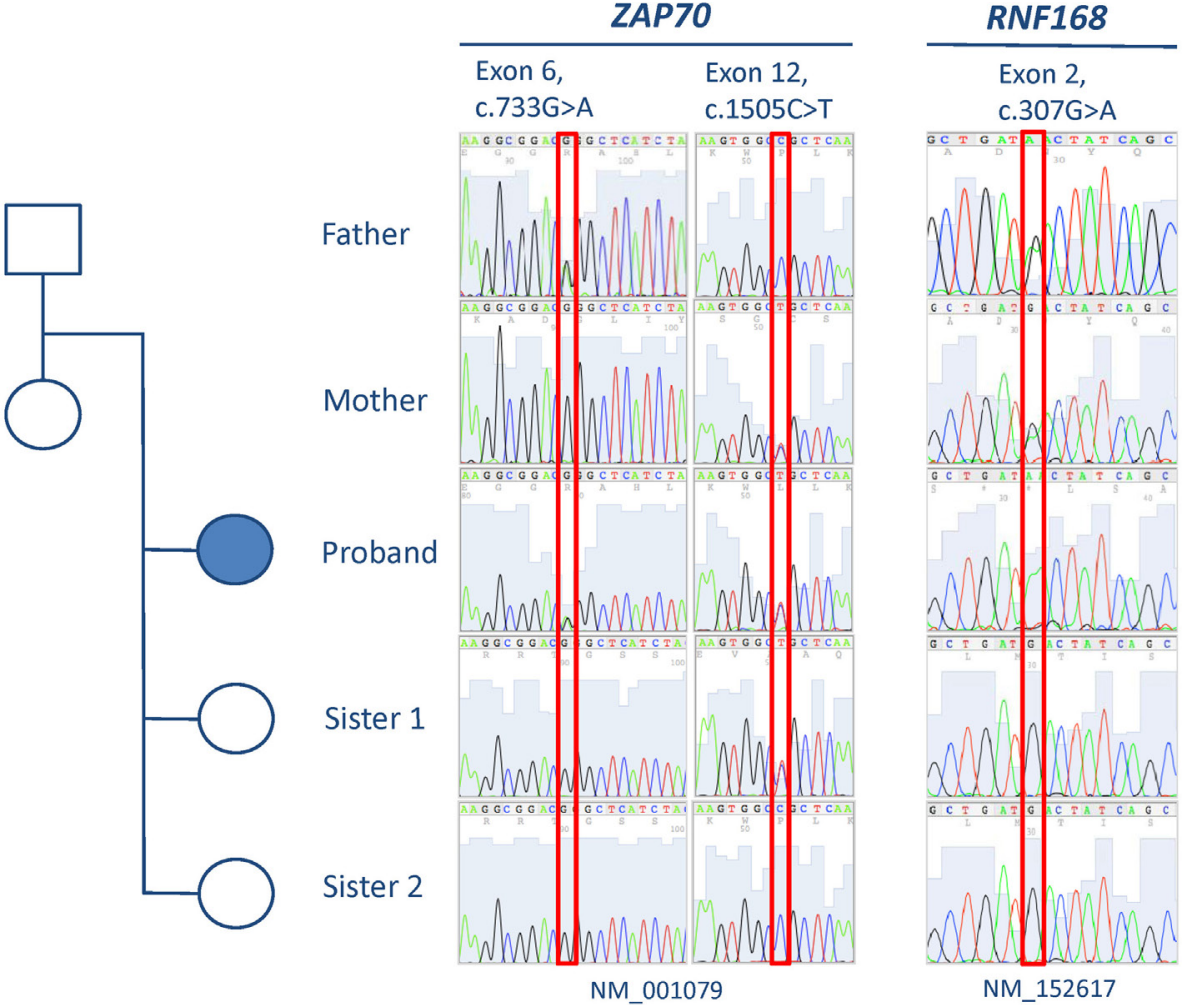
**Pedigree and results from Sanger sequencing of exons 6 and 12 of *ZAP70* and exon 2 of *RNF168***.

Analysis of the exome data revealed the presence of two novel compound heterozygous missense variants in *ZAP70*, one in exon 12 [c.1505C>T (NM_001079), p.P502L] and the other in exon 6 [c.733G>A (NM_001079), p.G245R], that could explain the immunologic defects present in the patient (Table 2). Familial cosegregation of the variants with phenotype was confirmed by Sanger sequencing (Figure 1). The mother was a heterozygous carrier of the exon 12 variant, and the father was a heterozygous carrier of the exon 6 variant. Two healthy sisters were also tested, and one was found to be a heterozygous carrier of the exon 12 variant. Both variants affect highly conserved residues and lead to amino acid changes that are predicted to be damaging by Combined Annotation Dependent Depletion (CADD) score (34 for c.1505C>T and 33 for c.733G>A) (14–16). No other variants were observed that could provide a suitable explanation for the T cell defects (Table S1 in Supplementary Material).

**Table 2:** **Variants relevant to phenotype**.

Gene	Coordinates	Zygosity	Variant reads	Total reads	ExAC MAF	PhyloP score	CADD Phred score	Exon	cDNA change	Protein change
**Variants of interest**										
*ZAP70*	Chr2:98349618_G>A	Het	54	112	0.00003	0.9996 (C)	33	6	c.733G>A	p.G245R
*ZAP70*	Chr2:98354242_C>T	Het	36	64	0	0.9987 (C)	34	12	c.1505C>T	p.P502L
*RNF168*	Chr3:196215549_C>T	Hom	10	10	0.02	0.9837 (C)	15.07	2	c.307G>A	p.D103N

**Protein-altering variants in other double-strand break DNA repair genes^a^ (ExAC MAF ≤0.05, PhyloP prediction = conserved, CADD Phred score ≥15)**										

*MLH1*	Chr3:37092019_G>A	Het	55	101	0.001	0.9994 (C)	24.6	19	c.2146G>A	p.V716M

*^a^Genes examined: APE1, APTX, ATM, ATMIN, BLM, BRCA1, C21ORF2, C9ORF142, CDKN1A, CHD1, DCLRE1C, DKC1, ERCC2, EXO1, FAM65B, FANCD2, HDM2, HSPBAP1, IL13RA1, INSIG1, IP6K2, KAT7, KDM4B, LIG3, LIG4, MDC1, MGA, MIR34A, MLH1, MRE11A, MSH2, MSH6, NABP2, NBN, NCK1, NHEJ1, PARP1, PARP10, PMS2, POLM, PRKDC, RAD9A, RAD50, RIC8B, RIF1, RNF168, SIRT1, SMC1A, TERC, TGFB1, TP53, TP53BP1, TSPAN12, UBA2, XRCC1, XRCC2, XRCC3, XRCC4, XRCC5, XRCC6, ZEB1. Genes associated with radiosensitivity in OMIM are underlined*.

*C, conserved; Het, heterozygous; Hom, homozygous; MAF, minor allele frequency*.

**Figure 2:**
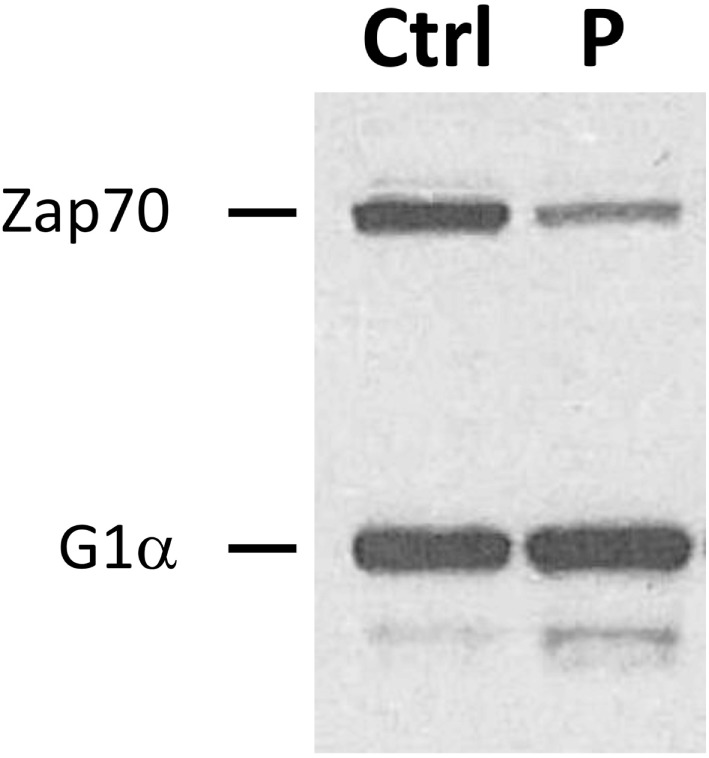
**Reduced expression of ZAP70 protein**. Immunoblotting was performed to detect ZAP70 in lysates obtained from patient (P) and control (Ctrl) peripheral blood lymphocytes. Simultaneous assessment of G1α expression is shown as a loading control.

Subsequent investigations of lymphocytes isolated from the patient supported the presence of ZAP70 deficiency. *In vitro* proliferation was depressed at 10% [stimulation index (SI) = 18] of control responses (SI = 196) to phytohemagglutinin. Proliferation after stimulation with anti-CD3 antibody was similarly impaired (patient SI = 18, control SI = 156). The response was partially rescued by incubation with phorbol 12-myristate 13-acetate and ionomycin, as previously observed in other patients with ZAP70 deficiency (5). T cell receptor excision circle levels were also extremely low (47 per 0.5 µg of DNA, normal >400) (17). Reduced ZAP70 protein expression was demonstrated in patient lymphocytes by immunoblotting (Figure 2) (18). Phosphorylation of ZAP70 was diminished after co-incubation of patient lymphocytes with anti-CD3 antibody (results not shown), although it remains unclear whether this finding resulted from the overall decreased ZAP70 expression, inability to auto-phosphorylate, or both.

Further analyses were then performed to identify a genetic cause for the radiation sensitivity. We examined the 21,536 variants identified by WES for 61 genes (Table 2) encoding molecules known or anticipated to be associated with a cellular phenotype of radiosensitivity containing protein-altering (truncating, splicing, or non-synonymous) changes. Non-synonymous variants were considered if predicted to be conserved (by PhyloP) and damaging (CADD score ≥15) (14, 16). Only one variant with an ExAC minor allelic frequency (MAF) below 0.01 was identified in this manner: a single heterozygous missense change in *MLH1* [c.2146G>A (NM_000249), p.V716M]. While biallelic *MLH1* mutations are associated with slight radiosensitivity in cultured skin fibroblasts (Mendelian Inheritance in Man #276300), single allelic variants in the gene are not known to produce this phenotype. As a result and because polymorphisms have been reported to be associated with radiosensitivity, we expanded the analyses to identify variants in the same 61 genes with MAFs below 0.05 (19–22). This analysis revealed only the presence of a homozygous missense variant in exon 2 of *RNF168* [c.307G>A, (NM_152617), p.D103N] (Figure 1). Both parents were confirmed to be heterozygous carriers of the *RNF168* variant, but neither of the two siblings received a copy of the altered gene (Figure 1).

## Background

### History

ZAP70 deficiency was first reported in 1994 (5, 23, 24). ZAP70 plays a critical role in T cell receptor signal transduction (5, 25). As a result, biallelic mutations of *ZAP70* are known to produce both abnormal thymic development of T cells and defective T cell function (5, 26). Because ZAP70 is highly expressed in CD8^+^ T cells and NK cells, loss of function can lead to recurrent and severe viral infections.

Radiosensitivity, immunodeficiency, dysmorphic features, and learning difficulties (RIDDLE) syndrome (Mendelian Inheritance in Man #611943) was first described in 2007 (27). The condition was ultimately determined to be caused by biallelic mutations in *RNF168* (27, 28). RNF168 is a ubiquitin-ligase ring-finger protein that plays an important role in the ubiquitin-dependent DNA damage response (9, 29). After recognition of a double-stranded DNA break by ATM, MRE11, RAD50, and nibrin, RNF168 is recruited to the site by mono- or di-ubiquitinated histone H2A motifs. In addition, in combination with UBC13, it also directly ubiquitinates H2A during this process. RNF168 then extends the histone modifications, forming polyubiquitin chains to recruit BRCA1 and 53BP1, which facilitate DNA repair.

### Review of Similar Cases

ZAP70 deficiency results in a spectrum of immune deficiency or dysregulation ranging from atopy, autoimmunity, and late-onset combined immunodeficiency to severe, combined T and B cell deficiency from birth (6, 24, 30, 31). Many mutations have been identified in the gene, impacting the protein in several domains (Figure S1 in Supplementary Material, as reported in the Human Gene Mutation Database and Mendelian Inheritance in Man). Complete deficiency of protein expression results in severe immune deficiency that results in marked susceptibility to infections within the first few months of life (26). Hypomorphic mutations produce overall T cell lymphopenia, reduced T cell function, and later-onset presentation of clinical disease, as seen in our patient (26, 31). In all cases, CD8^+^ T cell lymphopenia is present.

Two patients with *RNF168* deficiency have been reported to date (27, 32). In the first, immune deficiency was characterized by low serum IgG levels (27). The other patient had IgA deficiency but normal serum IgG levels (32). Both patients exhibited infectious susceptibility in a similar manner to our patient. In RNF168-deficient mice, the immunologic defects are more pronounced (33). The first patient was reported to have learning difficulties, but the second had normal intelligence. Our patient currently lives with her parents but functions independently. She recently completed a bachelor’s degree in art and psychology. Both previously reported individuals also had short stature, motor deficits or ataxia, dysmorphic facial features or microcephaly, and radiosensitivity. Of these additional features, our patient exhibited only radiosensitivity. That said, her severe radiation hypersensitivity suggests that the detected *RNF168* variant may be phenotypically relevant and impactful. The mutations in the 2 previously reported patients are highly deleterious (compound heterozygous frameshift changes leading to premature stop codons in the first patient and a homozygous p.R131X mutation in the second, see Figure S1 in Supplementary Material), perhaps accounting for the severe phenotypes. The less damaging homozygous missense variant in our patient may produce a milder phenotype consisting only of radiation sensitivity.

## Discussion

### Diagnosis

Recent advances in our understanding of the genetics and pathophysiology of immune deficiency have shown that mutations in critical pathways in T cell development and function can result in a broad spectrum of clinical phenotypes. The presented patient is, to our knowledge, not only the oldest reported patient with newly diagnosed ZAP70 deficiency but also expresses a unique blended clinical phenotype and genotype with mutations linked to two distinct immune deficiency disorders. Diagnosis of ZAP70 deficiency in adulthood is unusual, and it remains unclear whether the homozygous *RNF168* variant may have altered the classic presentation and course of ZAP70 deficiency in manners that we do not yet understand. *Vice versa*, the possibility that ZAP70 deficiency may have affected the biological impact of the *RNF168* homozygous variant cannot be excluded. The relatively high allelic frequency of this *RNF168* variant (0.02 in the ExAC database), especially among the Hispanic population (0.19), may suggest that radiosensitivity represents a clinical phenotype that occurs more commonly than recognized, perhaps because it is rarely tested. Indeed, it has been recognized that 20–40% of apparently healthy individuals have increased radiosensitivity and impaired DNA damage repair when screened for these abnormalities (34–37). Thus, it seems possible that homozygous carriers of the *RNF168* c.307G>A variant typically possess radiation sensitivity that is not clinically apparent. As such, this variant may have clinical implications, especially if it represents a cancer susceptibility gene defect. Meanwhile, ZAP70 is known to be expressed in B cells, although its function in these cells remains poorly understood (38–40). If it has a role in B cell survival, particularly after a critical insult, such as ionizing radiation-induced DNA damage, loss of ZAP70 function could result in augmented radiation sensitivity, as assessed by the clinical *in vitro* DNA repair disorder assay. This hypothesis merits further future investigations.

### Treatment

With the identification of the variants in *ZAP70* and *RNF168*, the patient has been referred for evaluation for potentially curative allogeneic hematopoietic stem cell transplantation (HSCT). Elements favoring this approach include the poor quality of life (due to the skin problems and significant medical support consisting of IgG supplementation; antibiotic, antifungal, and antiviral therapy or prophylaxis; and IFN-γ for HPV infection) and continued oncologic risk. On the other hand, this patient has survived into the third decade of life. While allogeneic HSCT is recommended to occur as early as possible for children with *ZAP70* deficiency to prevent fatal infectious complications, the current age of the patient makes the potential survival benefit from allogeneic HSCT less clear (41). In addition, she has considerable transplantation risks, including suppressed HPV and cryptococcal infections, absence of a fully HLA-matched donor, active skin inflammation that may increase the risk of skin graft-versus-host disease, radiation sensitivity, and advanced age.

## Concluding Remarks

In summary, we report the oldest individual diagnosed with ZAP70-related primary immune deficiency. In addition, through WES, we were able to identify the presence of potentially pathogenic mutant alleles at two loci, resulting in a blended phenotype. Blended phenotypes have been reported to occur in about 5–7% of individuals tested by WES with a higher incidence (11%) in patients with PIDDs (1, 42–45). In this patient, WES demonstrated compound heterozygosity for two novel mutations in *ZAP70* and homozygosity for a missense variant *RNF168*, which may confer radiation sensitivity. The finding of sequence alterations in two distinct disease genes with a blended clinical phenotype of combined immunodeficiency and radiation hypersensitivity has not been reported in the literature. This case highlights the importance of examining for mutations in multiple loci beyond focusing upon single likely candidate genes. It strongly suggests the utility of WES and comprehensive and genomic testing for patients with PIDDs, particularly for patients who exhibit atypical clinical features, to exclude the presence of additional molecular defects that may have implications concerning various approaches for therapy (1). As such, multilocus genomic variation and the coexistence of two underlying genotypic abnormalities in this individual also draw attention to the clinical complexity of managing patients with such distinct or overlapping molecular phenotypes (45).

## Ethics Statement

Written informed consent was obtained from the patient and her family members to participate in a Baylor College of Medicine Institutional Review Board approved protocol for WES as part of the Baylor-Hopkins Center for Mendelian Genomics project at Baylor College of Medicine (Houston, TX, USA). The patient and family members also provided written informed consent to have their clinical and genetic information published in medical or scientific journals. All procedures performed in studies involving human participants were in accordance with the ethical standards of the institutional and/or national research committee and with the 1964 Helsinki declaration and its later amendments or comparable ethical standards.

## Author Contributions

IC collected data, performed genetic data analyses, and wrote the manuscript. RS collected clinical data and prepared a draft of the manuscript. AS-P collected and analyzed genetic data. ZC-A provided bioinformatics for genetic data analyses. VK collected samples. HD performed biological testing. CR provided supervision of research. TQ collected clinical data and prepared a draft of the manuscript. JL provided WES and genomic and genetic data analyses and wrote portions of the manuscript. JO provided supervision of the clinical care and research. IH collected clinical and immunologic data and wrote portions of the manuscript.

## Conflict of Interest Statement

Baylor College of Medicine (BCM) and Miraca Holdings, Inc. have formed a joint venture with shared ownership and governance of Baylor Genetics Laboratories (BG), which performs clinical exome sequencing. JL derives support through a professional services agreement between BCM and BG. JL also serves on the Scientific Advisory Board of the BG. JL has stock ownership in 23andMe, is a paid consultant for Regeneron Pharmaceuticals, has stock options in Lasergen, Inc., and is a coinventor of US and European patents related to molecular diagnostics for inherited neuropathies, eye diseases, and bacterial genomic fingerprinting. None of the remaining authors have potential conflicts of interest.

## Supplementary Material

Figure S1 **Known mutations in the (A) ZAP70 and (B) RNF168 proteins, as reported in the Human Gene Mutation and Mendelian Inheritance in Man databases**. Amino acid positions are marked above and mutations are shown below each protein using dotted lines. **(A)** ZAP70 protein domains are labeled within the shaded boxes. Patient mutations are designated by arrows above the protein. Note that the figure does not include the known IVS7 c.837 + 121G>A, IVS12 c.1624-11G>A, and c. 836_837delAT mutations. **(B)** RNF168 protein domains are labeled above the amino acid positions. Abbreviations: RING, really interesting new gene finger; LRM, LR motif; MIU, motif interacting with ubiquitin; UMI, ubiquitin interacting motif- and MIU-related ubiquitin binding domain. Patient mutations are designated by arrows below the protein.

Table S1 **Other proband variants identified by WES**.

## References

[1] Stray-Pedersen A Sorte HS Samarakoon P Gambin T Chinn IK Coban Akdemir ZH Primary immunodeficiency diseases: genomic approaches delineate heterogeneous Mendelian disorders. J Allergy ClinImmunol (2017) 139(1):232–45. 10.1016/j.jaci.2016.05.042 PMC5222743 27577878

[2] Fleisher TA Bosco Oliveira J. Functional and molecular evaluation of lymphocytes. J Allergy ClinImmunol (2004) 114(2):227–34. 10.1016/j.jaci.2004.06.001 15316494

[3] Huo YK Wang Z Hong J-H Chessa L McBride WH Perlman SL Radiosensitivity of ataxia-telangiectasia, X-linked agammaglobulinemia, and related syndromes using a modified colony survival assay. Cancer Res (1994) 54(10):2544–7. 8168076

[4] Sun X Becker-Catania SG Chun HH Hwang MJ Huo Y Wang Z Early diagnosis of ataxia-telangiectasia using radiosensitivity testing. J Pediatr (2002) 140(6):724–31. 10.1067/mpd.2002.123879 12072877

[5] Arpaia E Shahar M Dadi H Cohen A Rolfman CM. Defective T cell receptor signaling and CD8+ thymic selection in humans lacking Zap-70 kinase. Cell (1994) 76(5):947–58. 10.1016/0092-8674(94)90368-9 8124727

[6] Turul T Tezcan I Artac H de Bruin-Versteeg S Barendregt BH Reisli I Clinical heterogeneity can hamper the diagnosis of patients with ZAP70 deficiency. Eur J Pediatr (2008) 168(1):87–93. 10.1007/s00431-008-0718-x 18509675

[7] Mazer B Harbeck RJ Franklin R Schwinzer R Kubo R Hayward A Phenotypic features of selective T cell deficiency characterized by absence of CD8+T lymphocytes and undetectable mRNA for ZAP-70 kinase. Clin Immunol Immunopathol (1997) 84(2):129–38. 10.1006/clin.1997.4365 9245543

[8] Chinn IK Shearer WT Severe combined immunodeficiency disorders. Immunol Allergy Clin North Am (2015) 35(4):671–94. 10.1016/j.iac.2015.07.002 26454313

[9] Blundred RM Stewart GS. DNA double-strand break repair, immunodeficiency and the RIDDLE syndrome. Expert Rev Clin Immunol (2011) 7(2):169–85. 10.1586/eci.10.93 21426255

[10] Gatti RA Boder E Good RA Immunodeficiency, radiosensitivity, and the XCIND syndrome. Immunol Res (2007) 38(1):87–101. 10.1007/s12026-007-0018-y 17917014

[11] Nahas SA Gatti RA DNA double strand break repair defects, primary immunodeficiency disorders, and ‘radiosensitivity’. Curr Opin Allergy Clin Immunol (2009) 9(6):510–6. 10.1097/ACI.0b013e328332be17 19858715

[12] Mizutani S Takagi M. XCIND as a genetic disease of X-irradiation hypersensitivity and cancer susceptibility. Int J Hematol (2013) 97(1):37–42. 10.1007/s12185-012-1240-5 23266960

[13] Lupski JR Gonzaga-Jauregui C Yang Y Bainbridge MN Jhangiani S Buhay CJ Exome sequencing resolves apparent incidental findings and reveals further complexity of SH3TC2 variant alleles causing Charcot-Marie-Tooth neuropathy. Genome Med (2013) 5(6):57. 10.1186/gm461 23806086 PMC3706849

[14] Pollard KS Hubisz MJ Rosenbloom KR Siepel A. Detection of nonneutral substitution rates on mammalian phylogenies. Genome Res (2010) 20(1):110–21. 10.1101/gr.097857.109 19858363 PMC2798823

[15] Kircher M Witten DM Jain P O’Roak BJ Cooper GM Shendure J. A general framework for estimating the relative pathogenicity of human genetic variants. Nat Genet (2014) 46(3):310–5. 10.1038/ng.2892 24487276 PMC3992975

[16] Meyts I Bosch B Bolze A Boisson B Itan Y Belkadi A Exome and genome sequencing for inborn errors of immunity. J Allergy ClinImmunol (2016) 138(4):957–69. 10.1016/j.jaci.2016.08.003 27720020 PMC5074686

[17] Roifman CM Dadi H Somech R Nahum A Sharfe N Characterization of ζ-associated protein, 70 kd (ZAP70)-deficient human lymphocytes. J Allergy ClinImmunol (2010) 126(6):1226.e–33.e. 10.1016/j.jaci.2010.07.029 20864151

[18] Dadi HK Simon AJ Roifman CM Effect of CD3δ deficiency on maturation of α/β and γ/δ T-cell lineages in severe combined immunodeficiency. N Engl J Med (2003) 349(19):1821–8. 10.1056/NEJMoa031178 14602880

[19] Smirnov DA Brady L Halasa K Morley M Solomon S Cheung VG. Genetic variation in radiation-induced cell death. Genome Res (2012) 22(2):332–9. 10.1101/gr.122044.111 21844125 PMC3266040

[20] Hornhardt S Rößler U Sauter W Rosenberger A Illig T Bickeböller H Genetic factors in individual radiation sensitivity. DNA Repair (2014) 16:54–65. 10.1016/j.dnarep.2014.02.001 24674628

[21] Fuentes-Raspall MJ Caragol I Alonso C Ramón y Cajal T Fisas D Seoane A Apoptosis for prediction of radiotherapy late toxicity: lymphocyte subset sensitivity and potential effect of TP53 Arg72Pro polymorphism. Apoptosis (2015) 20(3):371–82. 10.1007/s10495-014-1056-2 25398538

[22] Alsbeih G Al-Meer RS Al-Harbi N Bin Judia S Al-Buhairi M Venturina NQ Gender bias in individual radiosensitivity and the association with genetic polymorphic variations. Radiother Oncol (2016) 119(2):236–43. 10.1016/j.radonc.2016.02.034 26987471

[23] Chan AC Kadlecek TA Elder ME Filipovich AH Kuo WL Iwashima M ZAP-70 deficiency in an autosomal recessive form of severe combined immunodeficiency. Science (1994) 264(5165):1599. 10.1126/science.8202713 8202713

[24] Elder ME Lin D Clever J Chan AC Hope TJ Weiss A Human severe combined immunodeficiency due to a defect in ZAP-70, a T cell tyrosine kinase. Science (1994) 264(5165):1596–9. 10.1126/science.8202712 8202712

[25] Chan AC Iwashima M Turck CW Weiss A ZAP-70: a 70 kd protein-tyrosine kinase that associates with the TCR zeta chain. Cell (1992) 71(4):649–62. 10.1016/0092-8674(92)90598-7 1423621

[26] Fischer A Picard C Chemin K Dogniaux S le Deist F Hivroz C. ZAP70: a master regulator of adaptive immunity. Semin Immunopathol (2010) 32(2):107–16. 10.1007/s00281-010-0196-x 20135127

[27] Stewart GS Stankovic T Byrd PJ Wechsler T Miller ES Huissoon A RIDDLE immunodeficiency syndrome is linked to defects in 53BP1-mediated DNA damage signaling. Proc Natl Acad Sci U S A (2007) 104(43):16910–5. 10.1073/pnas.0708408104 17940005 PMC2040433

[28] Stewart GS Panier S Townsend K Al-Hakim AK Kolas NK Miller ES The RIDDLE syndrome protein mediates a ubiquitin-dependent signaling cascade at sites of DNA damage. Cell (2009) 136(3):420–34. 10.1016/j.cell.2008.12.042 19203578

[29] Hiom K. DNA repair: a riddle at a double-strand break. Curr Biol (2009) 19(8):R331–3. 10.1016/j.cub.2009.03.024 19409282

[30] Rosenberg SL Larkin A ZAP70-related severe combined immunodeficiency. In: Pagon RA Adam MP Ardinger HH Wallace SE Amemiya A Bean LJH, editors. GeneReviews. Seattle, WA: University of Washington (2014). p. 1993–2015.

[31] Picard C Dogniaux S Chemin K Maciorowski Z Lim A Mazerolles F Hypomorphic mutation of ZAP70 in human results in a late onset immunodeficiency and no autoimmunity. Eur J Immunol (2009) 39(7):1966–76. 10.1002/eji.200939385 19548248

[32] Devgan SS Sanal O Doil C Nakamura K Nahas SA Pettijohn K Homozygous deficiency of ubiquitin-ligase ring-finger protein RNF168 mimics the radiosensitivity syndrome of ataxia-telangiectasia. Cell Death Differ (2011) 18(9):1500–6. 10.1038/cdd.2011.18 21394101 PMC3178430

[33] Bohgaki T Bohgaki M Cardoso R Panier S Zeegers D Li L Genomic instability, defective spermatogenesis, immunodeficiency, and cancer in a mouse model of the riddle syndrome. PLoS Genet (2011) 7(4):e1001381 10.1371/journal.pgen.1001381 21552324 PMC3084200

[34] Marcon F Andreoli C Rossi S Verdina A Galati R Crebelli R. Assessment of individual sensitivity to ionizing radiation and DNA repair efficiency in a healthy population. Mutat Res (2003) 541(1–2):1–8. 10.1016/S1383-5718(03)00171-2 14568289

[35] Sigurdson AJ Stram DO. Genetic predisposition to radiation-related cancer and potential implications for risk assessment. Ann ICRP (2012) 41(3–4):108–16. 10.1016/j.icrp.2012.06.030 23089009 PMC3916824

[36] Kato TA Wilson PF Nagasaw H Peng Y Weil MM Little JB Variations in radiosensitivity among individuals: a potential impact on risk assessment? Health Phys (2009) 97(5):470–80. 10.1097/HP.0b013e3181b08eee 19820456

[37] Hu JJ Smith TR Miller MS Lohman K Case LD. Genetic regulation of ionizing radiation sensitivity and breast cancer risk. Environ Mol Mutagen (2002) 39(2–3):208–15. 10.1002/em.10058 11921191

[38] Anbazhagan K Rabbind Singh A Isabelle P Stella I Céline A-DM Bissac E Human pre-B cell receptor signal transduction: evidence for distinct roles of PI3kinase and MAP-kinase signalling pathways. Immun Inflamm Dis (2013) 1(1):26–36. 10.1002/iid3.4 25400915 PMC4217539

[39] Fedele AL Tolusso B Gremese E Bosello SL Carbonella A Canestri S Memory B cell subsets and plasmablasts are lower in early than in long-standing rheumatoid arthritis. BMC Immunol (2014) 15(1):28. 10.1186/s12865-014-0028-1 25187226 PMC4168163

[40] Michelutti A Gremese E Morassi F Petricca L Arena V Tolusso B B-cell subsets in the joint compartments of seropositive and seronegative rheumatoid arthritis (RA) and no-RA arthritides express memory markers and ZAP70 and characterize the aggregate pattern irrespectively of the autoantibody status. Mol Med (2011) 17(9–10):901–9. 10.2119/molmed.2011.00034 21607290 PMC3188870

[41] Kim VH-D Murguia L Schechter T Grunebaum E Roifman CM Emergency treatment for ζ chain-associated protein of 70 kDa (ZAP70) deficiency. J Allergy ClinImmunol (2013) 131(4):1233–5. 10.1016/j.jaci.2012.09.020 23141738

[42] Yang Y Muzny DM Reid JG Bainbridge MN Willis A Ward PA Clinical whole-exome sequencing for the diagnosis of Mendelian disorders. N Engl J Med (2013) 369(16):1502–11. 10.1056/NEJMoa1306555 24088041 PMC4211433

[43] Yang Y Muzny DM Xia F Niu Z Person R Ding Y Molecular findings among patients referred for clinical whole-exome sequencing. JAMA (2014) 312(18):1870–9. 10.1001/jama.2014.14601 25326635 PMC4326249

[44] Posey JE Rosenfeld JA James RA Bainbridge M Niu Z Wang X Molecular diagnostic experience of whole-exome sequencing in adult patients. Genet Med (2016) 18(7):678–85. 10.1038/gim.2015.142 26633545 PMC4892996

[45] Posey JE Harel T Liu P Rosenfeld JA James RA Coban Akdemir ZH Resolution of disease phenotypes resulting from multilocus genomic variation. N Engl J Med (2017) 376:21–31. 10.1056/NEJMoa1516767 27959697 PMC5335876

