# Supplementary material for: Novel Combined Immune Deficiency and Radiation Sensitivity Blended Phenotype in an Adult with Biallelic Variations in ZAP70 and RNF168
Source: Front Immunol. 2017 May 26;8:576. doi: 10.3389/fimmu.2017.00576 (PMC5445153; doi:10.3389/fimmu.2017.00576)
Supplement: Table S1 — Other proband variants identified by WES. [file table_1.doc]

**Supplemental Table.**

| *Gene* | *Coordinates* | *Variant reads* | *Total reads* | *Effect* | *Reference* | *Exon* | *cDNA change* | *Protein change* |
| --- | --- | --- | --- | --- | --- | --- | --- | --- |
| *Primary immunodeficiency disease-associated genes:* | | | | | | | | |
| RPS24 | 10:79814665_C>T | 24 | 46 | nonsynonymous | NM_001142285 | exon5 | c.C767T | p.P256L |
| CD55 | 1:207504593_G>A | 26 | 53 | nonsynonymous | NM_000574 | exon6 | c.G805A | p.V269M |
| CARD14 | 17:78177659_C>A | 68 | 115 | nonsynonymous | NM_024110 | exon16 | c.C2258A | p.T753N |
| ADA | 20:43251694_C>T | 23 | 43 | nonsynonymous | NM_000022 | exon7 | c.G632A | p.R211H |
| *Predicted primary immunodeficiency disease-associated genes:* | | | | | | | | |
| LCOR | 10:98667508_A>G | 20 | 51 | splicing | N/A | N/A | N/A | N/A |
| CCND1 | 11:69458595_C>T | 3 | 26 | splicing | NM_053056 | N/A | c.414-5C>T | N/A |
| CAMK1 | 3:9799310_A>G | 46 | 81 | splicing | NM_003656 | N/A | c.1030-5T>C | N/A |
| BMS1 | 10:43288435_G>C | 44 | 111 | nonsynonymous | NM_014753 | exon8 | c.G932C | p.S311T |
| IL6R | 1:154407585_G>A | 44 | 87 | nonsynonymous | NM_181359 | exon5 | c.G760A | p.E254K |
| ERBB3 | 12:56489585_G>C | 52 | 111 | nonsynonymous | NM_001982 | exon17 | c.G2050C | p.G684R |
| MAP3K6 | 1:27688031_G>C | 39 | 85 | nonsynonymous | NM_004672 | exon11 | c.C1666G | p.L556V |
| IFT88 | 13:21205180_A>C | 54 | 114 | nonsynonymous | NM_175605 | exon18 | c.A1352C | p.E451A |
| PRKD1 | 14:30099993_C>T | 34 | 72 | nonsynonymous | NM_002742 | exon10 | c.G1627A | p.V543I |
| FES | 15:91433098_G>A | 13 | 41 | nonsynonymous | NM_001143783 | exon6 | c.G781A | p.V261M |
| PRKCA | 17:64684457_G>T | 30 | 76 | nonsynonymous | NM_002737 | exon7 | c.G724T | p.V242L |
| RBL1 | 20:35651132_G>A | 48 | 84 | nonsynonymous | NM_002895 | exon17 | c.C2480T | p.T827I |
| BIN1 | 2:127811522_G>T | 105 | 192 | nonsynonymous | NM_139343 | exon13 | c.C1198A | p.P400T |
| COL18A1 | 21:46876025_C>T | 19 | 48 | nonsynonymous | NM_030582 | exon1 | c.C581T | p.S194L |
| CEP63 | 3:134278215_A>G | 48 | 96 | nonsynonymous | NM_025180 | exon15 | c.A1897G | p.N633D |
| MECOM | 3:168834305_T>G | 51 | 98 | nonsynonymous | NM_001105077 | exon8 | c.A986C | p.D329A |
| LTF | 3:46497407_G>C | 24 | 54 | nonsynonymous | NM_001199149 | exon4 | c.C246G | p.N82K |
| FGA | 4:155507509_C>T | 32 | 58 | nonsynonymous | NM_021871 | exon5 | c.G1072A | p.G358R |
| TGFBI | 5:135397231_C>T | 50 | 91 | nonsynonymous | NM_000358 | exon15 | c.C1949T | p.A650V |
| CDK19 | 6:110942426_C>T | 24 | 50 | nonsynonymous | NM_015076 | exon12 | c.G1258A | p.G420R |
| RAET1E | 6:150211179_T>C | 34 | 73 | nonsynonymous | NM_139165 | exon2 | c.A188G | p.Q63R |
| TEAD3 | 6:35445096_G>A | 13 | 35 | nonsynonymous | NM_003214 | exon8 | c.C584T | p.T195M |
| EPHB6 | 7:142561451_G>T | 5 | 19 | nonsynonymous | NM_004445 | exon6 | c.G163T | p.G55W |
| NOD1 | 7:30491421_G>T | 16 | 31 | nonsynonymous | NM_006092 | exon6 | c.C1612A | p.Q538K |
| BAG4 | 8:38067608_C>T | 52 | 101 | nonsynonymous | NM_004874 | exon5 | c.C971T | p.S324L |
| *Homozygous variants:* | | | | | | | | |
| ACOT1 | 14:74008343_AC>A | 151 | 157 | frameshift deletion | NM_001037161 | exon2 | c.605delC | p.T202fs |
| GSTM1 | 1:110233104_A>T | 836 | 839 | nonsynonymous | NM_000561 | exon7 | c.A485T | p.D162V |
| *Potentially compound heterozygous variants:* | | | | | | | | |
| FLG | 1:152283173_T>C | 36 | 141 | nonsynonymous | NM_002016 | exon3 | c.A4189G | p.N1397D |
| FLG | 1:152283175_G>C | 32 | 136 | nonsynonymous | NM_002016 | exon3 | c.C4187G | p.T1396S |
| FLG | 1:152283178_A>G | 35 | 138 | nonsynonymous | NM_002016 | exon3 | c.T4184C | p.V1395A |
| GAK | 4:843498_G>A | 24 | 39 | nonsynonymous | NM_005255 | exon28 | c.C3899T | p.S1300L |
| GAK | 4:882746_G>A | 14 | 29 | nonsynonymous | NM_005255 | exon11 | c.C1094T | p.A365V |
| ITIH5 | 10:7605146_T>C | 67 | 143 | nonsynonymous | NM_030569 | exon14 | c.A2729G | p.N910S |
| ITIH5 | 10:7621761_G>A | 21 | 54 | nonsynonymous | NM_030569 | exon9 | c.C1375T | p.R459W |
| QRICH2 | 17:74277983_C>T | 20 | 41 | nonsynonymous | NM_032134 | exon8 | c.G3727A | p.V1243I |
| QRICH2 | 17:74287186_C>T | 31 | 68 | nonsynonymous | NM_032134 | exon4 | c.G3124A | p.V1042M |
| SYNE2 | 14:64468654_T>A | 63 | 144 | nonsynonymous | NM_015180 | exon29 | c.T3641A | p.M1214K |
| SYNE2 | 14:64496634_G>A | 32 | 63 | nonsynonymous | NM_015180 | exon44 | c.G6736A | p.G2246S |
| SYNE2 | 14:64628873_C>T | 25 | 67 | nonsynonymous | NM_015180 | exon88 | c.C16178T | p.A5393V |
| *Single heterozygous loss of function variants:* | | | | | | | | |
| ZNF254 | 19:24309475_CT>C | 12 | 23 | frameshift deletion | NM_203282 | exon4 | c.674delT | p.L225fs |
| GBE1 | 3:81635338_CA>C | 14 | 44 | frameshift deletion | NM_000158 | exon10 | c.1239delT | p.D413fs |
| EXOC3L | 16:67220184_G>C | 34 | 70 | stopgain | NM_178516 | exon9 | c.C1452G | p.Y484X |
| ZNF717 | 3:75786985_C>A | 17 | 35 | stopgain | NM_001128223 | exon5 | c.G1789T | p.E597X |
| IL3RA | X:1467345_C>T | 232 | 465 | stopgain | NM_002183 | exon4 | c.C205T | p.Q69X |
| HBE1 | 11:5289832_T>C | 17 | 35 | splicing | NM_005330 | N/A | c.315-5A>G | N/A |
| ACACB | 12:109610165_G>A | 42 | 92 | splicing | NM_001093 | N/A | c.1117+4G>A | N/A |
| C12ORF29 | 12:88437494_TATC>T | 7 | 16 | splicing | N/A | N/A | N/A | N/A |
| FGD6 | 12:95500725_C>T | 13 | 29 | splicing | NM_018351 | N/A | c.3417+5G>A | N/A |
| MAN2C1 | 15:75659851_C>T | 21 | 55 | splicing | NM_006715 | N/A | c.351+1G>A | N/A |
| RNF123 | 3:49728560_C>T | 11 | 29 | splicing | N/A | N/A | N/A | N/A |
| CADPS | 3:62384023_T>C | 23 | 38 | splicing | N/A | N/A | N/A | N/A |
| TTC29 | 4:147861051_G>A | 31 | 56 | splicing | N/A | N/A | N/A | N/A |
| VPS37D | 7:73083745_G>A | 58 | 88 | splicing | NM_001077621 | N/A | c.138-4G>A | N/A |
| *Single heterozygous missense variants:* | | | | | | | | |
| VSX2 | 14:74726483_T>G | 21 | 44 | nonsynonymous | NM_182894 | exon4 | c.T758G | p.L253R |
| ADARB2 | 10:1313258_C>T | 17 | 40 | nonsynonymous | NM_018702 | exon4 | c.G1084A | p.A362T |
| GRIK4 | 11:120531034_C>T | 24 | 45 | nonsynonymous | NM_014619 | exon1 | c.C7T | p.R3C |
| IPO8 | 12:30827605_C>T | 31 | 67 | nonsynonymous | NM_006390 | exon7 | c.G812A | p.R271Q |
| KRT76 | 12:53170570_T>C | 67 | 135 | nonsynonymous | NM_015848 | exon1 | c.A506G | p.N169S |
| SPRYD3 | 12:53471014_T>A | 21 | 51 | nonsynonymous | NM_032840 | exon2 | c.A55T | p.N19Y |
| SCNN1A | 12:6464472_C>T | 79 | 197 | nonsynonymous | NM_001159576 | exon5 | c.G1286A | p.R429Q |
| DDHD1 | 14:53529781_C>T | 51 | 139 | nonsynonymous | NM_001160148 | exon7 | c.G1646A | p.G549D |
| TTC8 | 14:89307535_G>C | 40 | 70 | nonsynonymous | NM_198309 | exon4 | c.G454C | p.G152R |
| CYP4F22 | 19:15662138_G>C | 33 | 73 | nonsynonymous | NM_173483 | exon14 | c.G1452C | p.E484D |
| ANO8 | 19:17441012_G>T | 34 | 68 | nonsynonymous | NM_020959 | exon10 | c.C1195A | p.P399T |
| GTPBP5 | 20:60775817_G>A | 14 | 37 | nonsynonymous | NM_015666 | exon7 | c.G905A | p.R302H |
| RRP9 | 3:51969422_G>A | 25 | 38 | nonsynonymous | NM_004704 | exon10 | c.C907T | p.R303W |
| N4BP3 | 5:177546627_G>A | 41 | 91 | nonsynonymous | NM_015111 | exon2 | c.G43A | p.V15M |
| PTGER4 | 5:40681655_T>C | 19 | 37 | nonsynonymous | NM_000958 | exon2 | c.T560C | p.M187T |
| UBN2 | 7:138967900_T>C | 57 | 112 | nonsynonymous | NM_173569 | exon15 | c.T2249C | p.I750T |
| GALNT11 | 7:151800324_C>T | 57 | 100 | nonsynonymous | NM_022087 | exon4 | c.C547T | p.L183F |
| SRPX2 | X:99922291_G>A | 48 | 100 | nonsynonymous | NM_014467 | exon9 | c.G982A | p.V328I |
